# 2,2-Bis(3-chloro­methyl-4-ethoxy­phen­yl)propane

**DOI:** 10.1107/S1600536808018783

**Published:** 2008-06-28

**Authors:** Nejmeddine Jaballah, Taha Guerfel, Mustapha Majdoub

**Affiliations:** a Département de Chimie, Faculté des Sciences, 5019 Monastir, Tunisia

## Abstract

The title compound, C_21_H_26_Cl_2_O_2_, a bis-chloro­methyl derivative of *O*-ethyl­ated bis­phenol A, exhibits *C*_2_ mol­ecular symmetry. It shows a bent conformation with the two benzene rings nearly perpendicular [dihedral angle = 87.17 (6)°].

## Related literature

For more information on the synthesis, see: Miyazawa *et al.* (1999 ▶). For background to the investigation of new conjugated polymers derived from bis­phenols as potential organic semi-conducting materials, see: Jaballah *et al.* (2006 ▶). For the use of bis-chloro­methyl bis­phenol A ethers for the control of fungal and bacterial organisms, see: Priddy & Hennis (1970 ▶).
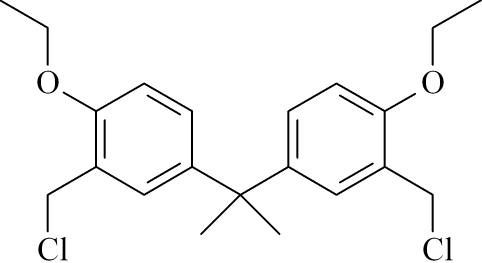


## Experimental

### 

#### Crystal data

C_21_H_26_Cl_2_O_2_
*M* *_r_* = 381.32
Monoclinic, 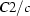

*a* = 13.856 (5) Å
*b* = 15.185 (6) Å
*c* = 10.999 (4) Å
β = 118.82 (3)°
*V* = 2027.7 (13) Å^3^
*Z* = 4
Mo *K*α radiation
μ = 0.33 mm^−1^
*T* = 293 (2) K
0.42 × 0.33 × 0.21 mm

#### Data collection

Enraf–Nonius TurboCAD-4 diffractometer
Absorption correction: none
2374 measured reflections
1960 independent reflections
1173 reflections with *I* > 2σ(*I*)
*R*_int_ = 0.022
2 standard reflections frequency: 120 min intensity decay: 2%

#### Refinement

*R*[*F* ^2^ > 2σ(*F* ^2^)] = 0.035
*wR*(*F* ^2^) = 0.118
*S* = 1.02
1960 reflections
166 parameters
H atoms treated by a mixture of independent and constrained refinement
Δρ_max_ = 0.25 e Å^−3^
Δρ_min_ = −0.21 e Å^−3^

### 

Data collection: *CAD-4 EXPRESS* (Enraf–Nonius, 1994 ▶); cell refinement: *CAD-4 EXPRESS*; data reduction: *XCAD4* (Harms & Wocadlo, 1995 ▶); program(s) used to solve structure: *SHELXS97* (Sheldrick, 2008 ▶); program(s) used to refine structure: *SHELXL97* (Sheldrick, 2008 ▶); molecular graphics: *ORTEP-3 for Windows* (Farrugia, 1997 ▶); software used to prepare material for publication: *WinGX* (Farrugia, 1999 ▶).

## Supplementary Material

Crystal structure: contains datablocks global, I. DOI: 10.1107/S1600536808018783/kp2175sup1.cif

Structure factors: contains datablocks I. DOI: 10.1107/S1600536808018783/kp2175Isup2.hkl

Additional supplementary materials:  crystallographic information; 3D view; checkCIF report

## Figures and Tables

**Fig. 1.**
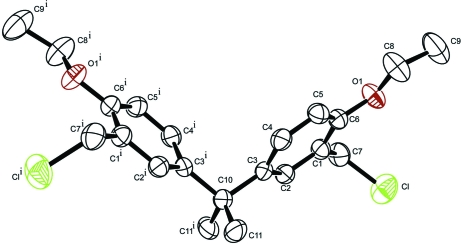
The molecular structure of BPAEtCl, showing the atom-labelling scheme. Displacement ellipsoids are drawn at the 50% probability level. H atoms have been omitted. Symmetry code: (i) -x + 1, y, -z + 5/2.

## supplementary crystallographic information

### Comment

BPAEtCl was synthesized as part of an ongoing program on the investigation of new conjugated polymers derived from bisphenols as potential organic semi-conducting materials (Jaballah *et al.*, 2006). This intermediate is of value in synthetic work inasmuch as the CH_2_C1 group can be converted to other groups such as CH_2_CN, CH_2_OH and CHO. Particularly, the bend-like structure of bisphenol A (BPA) nucleus offers a special interest in metacyclophanes synthesis (Miyazawa *et al.*, 1999). Bis-chloromethyl bisphenol A ethers are also useful as microbicides for control of fungal and bacterial organisms (Priddy & Hennis, 1970). The molecular structure of BPAEtCl is shown in Fig. 1. The two benzene  rings are  nearly perpendicular, forming a dihedral angle of 87.17 (6)°. The ethoxy group plan [O1—C8—C9] is almost parallel with the benzene ring with the dihedral angle of 6.82 (37)° whereas chloromethyl group plan [C1—C7—Cl] is close to be perpendicular [82.62 (13)°].

### Experimental

BPAEtCl was synthesized in two steps from 4,4'-isopropylidenediphenol [Bisphenol A, BPA]. To a stirred mixture of BPA (10 mmoles) and K_2_CO_3_ (40 mmoles) in 20 mL of dimethylformamide, was added dropwise bromoethane (30 mmoles). After stirring for 5 h at room temperature, the reaction mixture was poured into distilled water and extracted with diethyl ether. The extract was washed with distilled water, dried over anhydrous MgSO_4_, and then evaporated. The resultant crude product was purified by recrystallization from ethanol/water (3/1) to afford the 2,2-bis-(4-ethoxyphenyl)propane [BPAEt] as needle-like white crystals. A mixture of BPAEt (10 mmoles), paraformaldehyde (2.5 g), and 37% aqueous HCl (8.5 mL) in acetic acid (30 mL) was heated at 328 K for 5 h. The resulting mixture was then poured into distilled water and extracted with diethyl ether. The organic layer was washed several times with distilled water and dried over anhydrous MgSO_4_. After solvent removal and two recrystallizations from hexane, we obtained BPAEtCl as colourless crystals. Yield: 75%; mp: 352–354 K.

### Refinement

Hydrogen atoms were located in a fourier map and refined freely with isotropic thermal parameters.

### Crystal data

**Table:**

C_21_H_26_Cl_2_O_2_	*F*_000_ = 808
*M**_r_* = 381.32	*D*_x_ = 1.249 Mg m^−^^3^
Monoclinic, *C*2/*c*	Mo *K*α radiation λ = 0.71073 Å
Hall symbol: -C 2yc	Cell parameters from 25 reflections
*a* = 13.856 (5) Å	θ = 11.6–15.7º
*b* = 15.185 (6) Å	µ = 0.33 mm^−^^1^
*c* = 10.999 (4) Å	*T* = 293 (2) K
β = 118.82 (3)º	Prism, colourless
*V* = 2027.7 (13) Å^3^	0.42 × 0.33 × 0.21 mm
*Z* = 4	

### Data collection

**Table:**

Enraf–Nonius TurboCAD-4 diffractometer	*R*_int_ = 0.022
Radiation source: fine-focus sealed tube	θ_max_ = 26.0º
Monochromator: graphite	θ_min_ = 2.2º
*T* = 293(2) K	*h* = −17→17
non–profiled ω scans	*k* = −6→18
Absorption correction: none	*l* = −1→13
2374 measured reflections	2 standard reflections
1960 independent reflections	every 120 min
1173 reflections with *I* > 2σ(*I*)	intensity decay: 2%

### Refinement

**Table:**

Refinement on *F*^2^	H atoms treated by a mixture of independent and constrained refinement
Least-squares matrix: full	*w* = 1/[σ^2^(*F*_o_^2^) + (0.052*P*)^2^ + 0.7485*P*] where *P* = (*F*_o_^2^ + 2*F*_c_^2^)/3
*R*[*F*^2^ > 2σ(*F*^2^)] = 0.036	(Δ/σ)_max_ < 0.001
*wR*(*F*^2^) = 0.118	Δρ_max_ = 0.25 e Å^−^^3^
*S* = 1.03	Δρ_min_ = −0.21 e Å^−^^3^
1960 reflections	Extinction correction: none
166 parameters	

### Special details

**Table:**

Geometry. All e.s.d.'s (except the e.s.d. in the dihedral angle between two l.s. planes) are estimated using the full covariance matrix. The cell e.s.d.'s are taken into account individually in the estimation of e.s.d.'s in distances, angles and torsion angles; correlations between e.s.d.'s in cell parameters are only used when they are defined by crystal symmetry. An approximate (isotropic) treatment of cell e.s.d.'s is used for estimating e.s.d.'s involving l.s. planes.
Refinement. Refinement of *F*^2^ against ALL reflections. The weighted *R*-factor *wR* and goodness of fit *S* are based on *F*^2^, conventional *R*-factors *R* are based on *F*, with *F* set to zero for negative *F*^2^. The threshold expression of *F*^2^ > σ(*F*^2^) is used only for calculating *R*-factors(gt) *etc*. and is not relevant to the choice of reflections for refinement. *R*-factors based on *F*^2^ are statistically about twice as large as those based on *F*, and *R*- factors based on ALL data will be even larger.

### Fractional atomic coordinates and isotropic or equivalent isotropic displacement parameters (Å^2^)

**Table:**

	*x*	*y*	*z*	*U*_iso_*/*U*_eq_	
HC2	0.2827 (17)	0.0620 (14)	1.052 (2)	0.046 (6)*	
HC4	0.5740 (19)	0.1686 (14)	1.147 (2)	0.050 (6)*	
H111	0.641 (2)	0.0223 (19)	1.220 (3)	0.085 (9)*	
H211	0.6149 (19)	−0.0548 (15)	1.306 (3)	0.052 (6)*	
H1C7	0.124 (2)	0.1193 (16)	0.880 (3)	0.064 (8)*	
HC5	0.4965 (18)	0.2588 (15)	0.961 (2)	0.051 (6)*	
H311	0.5386 (19)	−0.0493 (15)	1.142 (3)	0.056 (6)*	
H2C7	0.126 (2)	0.2044 (18)	0.786 (3)	0.077 (8)*	
H2C8	0.403 (3)	0.278 (2)	0.719 (3)	0.102 (11)*	
H1C9	0.318 (3)	0.390 (2)	0.568 (4)	0.109 (13)*	
H2C9	0.217 (3)	0.403 (3)	0.598 (4)	0.128 (14)*	
H3C9	0.224 (4)	0.315 (3)	0.524 (5)	0.152 (17)*	
H1C8	0.392 (3)	0.362 (2)	0.807 (4)	0.107 (12)*	
Cl	0.11729 (5)	0.08024 (5)	0.67435 (7)	0.0780 (3)	
C10	0.5	0.04410 (18)	1.25	0.0424 (7)	
O1	0.28391 (12)	0.26525 (10)	0.77132 (17)	0.0557 (5)	
C3	0.43999 (15)	0.10349 (12)	1.1219 (2)	0.0360 (5)	
C1	0.27565 (15)	0.15480 (13)	0.9159 (2)	0.0395 (5)	
C5	0.45100 (18)	0.21933 (13)	0.9770 (2)	0.0436 (5)	
C2	0.32750 (16)	0.10062 (13)	1.0324 (2)	0.0386 (5)	
C4	0.49939 (17)	0.16456 (13)	1.0907 (2)	0.0420 (5)	
C6	0.33857 (16)	0.21428 (12)	0.8875 (2)	0.0408 (5)	
C7	0.15422 (18)	0.14840 (18)	0.8252 (3)	0.0522 (6)	
C11	0.5796 (2)	−0.01497 (17)	1.2272 (3)	0.0600 (8)	
C8	0.3483 (3)	0.3195 (2)	0.7315 (4)	0.0752 (9)	
C9	0.2741 (4)	0.3638 (3)	0.5969 (4)	0.0863 (11)	

### Atomic displacement parameters (Å^2^)

**Table:**

	*U*^11^	*U*^22^	*U*^33^	*U*^12^	*U*^13^	*U*^23^
Cl	0.0520 (4)	0.0956 (5)	0.0591 (5)	0.0027 (3)	0.0050 (3)	−0.0129 (4)
C10	0.0475 (15)	0.0365 (14)	0.0313 (17)	0	0.0095 (13)	0
O1	0.0532 (9)	0.0566 (9)	0.0505 (11)	0.0097 (7)	0.0195 (8)	0.0228 (8)
C3	0.0406 (10)	0.0330 (9)	0.0282 (12)	−0.0002 (7)	0.0116 (8)	−0.0029 (8)
C1	0.0371 (10)	0.0412 (10)	0.0358 (12)	0.0050 (8)	0.0141 (9)	0.0017 (9)
C5	0.0467 (11)	0.0390 (10)	0.0434 (14)	−0.0058 (9)	0.0204 (10)	0.0022 (10)
C2	0.0382 (10)	0.0382 (10)	0.0367 (13)	−0.0020 (8)	0.0159 (9)	−0.0001 (9)
C4	0.0358 (10)	0.0433 (11)	0.0363 (13)	−0.0055 (8)	0.0090 (9)	−0.0046 (9)
C6	0.0450 (11)	0.0383 (10)	0.0344 (13)	0.0055 (8)	0.0154 (9)	0.0030 (9)
C7	0.0400 (11)	0.0592 (14)	0.0478 (16)	0.0077 (10)	0.0135 (10)	0.0061 (12)
C11	0.0727 (17)	0.0472 (13)	0.0393 (16)	0.0191 (12)	0.0104 (13)	−0.0055 (12)
C8	0.0762 (19)	0.078 (2)	0.071 (2)	0.0090 (16)	0.0357 (17)	0.0314 (17)
C9	0.102 (3)	0.087 (2)	0.080 (3)	0.027 (2)	0.052 (2)	0.042 (2)

### Geometric parameters (Å, °)

**Table:**

Cl—C7	1.808 (3)	C5—HC5	0.95 (2)
C10—C11^i^	1.533 (3)	C2—HC2	0.95 (2)
C10—C11	1.533 (3)	C4—HC4	0.92 (2)
C10—C3^i^	1.537 (3)	C7—H1C7	0.99 (3)
C10—C3	1.537 (2)	C7—H2C7	0.95 (3)
O1—C6	1.368 (2)	C11—H111	1.06 (3)
O1—C8	1.430 (3)	C11—H211	0.97 (2)
C3—C2	1.386 (3)	C11—H311	0.98 (3)
C3—C4	1.388 (3)	C8—C9	1.494 (4)
C1—C6	1.392 (3)	C8—H2C8	1.04 (3)
C1—C2	1.395 (3)	C8—H1C8	1.00 (4)
C1—C7	1.489 (3)	C9—H1C9	0.91 (4)
C5—C4	1.377 (3)	C9—H2C9	0.99 (4)
C5—C6	1.387 (3)	C9—H3C9	1.07 (5)
C8—C9	1.494 (4)		
			
C11^i^—C10—C11	108.4 (3)	C1—C7—Cl	112.38 (17)
C11^i^—C10—C3^i^	107.90 (14)	C1—C7—H1C7	107.3 (15)
C11—C10—C3^i^	112.29 (13)	Cl—C7—H1C7	106.6 (15)
C11^i^—C10—C3	112.29 (13)	C1—C7—H2C7	109.6 (16)
C11—C10—C3	107.90 (14)	Cl—C7—H2C7	102.7 (17)
C3^i^—C10—C3	108.1 (2)	H1C7—C7—H2C7	118 (2)
C6—O1—C8	117.78 (18)	C10—C11—H111	111.7 (16)
C2—C3—C4	116.32 (18)	C10—C11—H211	108.1 (14)
C2—C3—C10	123.99 (17)	H111—C11—H211	109 (2)
C4—C3—C10	119.69 (16)	C10—C11—H311	109.7 (14)
C6—C1—C2	119.21 (18)	H111—C11—H311	109 (2)
C6—C1—C7	121.3 (2)	H211—C11—H311	109.5 (18)
C2—C1—C7	119.5 (2)	O1—C8—C9	109.2 (3)
C4—C5—C6	120.0 (2)	O1—C8—H2C8	107.3 (17)
C4—C5—HC5	118.4 (14)	C9—C8—H2C8	109.7 (19)
C6—C5—HC5	121.6 (14)	O1—C8—H1C8	110 (2)
C3—C2—C1	122.58 (19)	C9—C8—H1C8	113 (2)
C3—C2—HC2	119.3 (13)	H2C8—C8—H1C8	108 (3)
C1—C2—HC2	118.1 (13)	C8—C9—H1C9	107 (2)
C5—C4—C3	122.72 (19)	C8—C9—H2C9	115 (2)
C5—C4—HC4	118.3 (14)	H1C9—C9—H2C9	115 (3)
C3—C4—HC4	119.0 (14)	C8—C9—H3C9	109 (2)
O1—C6—C5	123.96 (19)	H1C9—C9—H3C9	110 (3)
O1—C6—C1	116.89 (18)	H2C9—C9—H3C9	101 (3)
C5—C6—C1	119.15 (19)		

Symmetry codes: (i) −*x*+1, *y*, −*z*+5/2.
